# Smaller dishware to reduce energy intake: fact or fiction?

**DOI:** 10.1186/s12966-019-0831-4

**Published:** 2019-08-28

**Authors:** Dana Lee Olstad, Clare Collins

**Affiliations:** 1 Department of Community Health Sciences, Cumming School of Medicine, University of Calgary, 3280 Hospital Drive NW, Calgary, Alberta T2N 4Z6 Canada; 2 School of Health Sciences, Faculty of Health and Medicine, Priority Research Centre in Physical Activity and Nutrition, ATC Building, University of Newcastle, Callaghan, New South Wales 2308 Australia

## Abstract

The potential effects of dishware size on energy intake are unclear, as many previous studies have been of low methodological quality. A newly published paper by Kosīte et al. (IJBNPA 10.1186/s12966-019-0826-1, 2019) reports findings from a rigorous, pre-registered investigation of the effects of manipulating plate size on total energy intake within a single eating occasion. This Editorial considers the implications of these new findings in light of previous evidence pertaining to the efficacy of behavioral nudges in particular, and in relation to contextual drivers of food consumption more generally. We conclude that the potential impact of behavioral nudges may have been exaggerated in the past, and call for future high-quality randomized controlled trials to establish whether reducing dishware size and other behavioral nudges might offer an effective complement to more comprehensive, multi-level interventions to reduce overconsumption of foods and beverages at a population-level.

Despite decades of focussed study and intervention, overconsumption of foods and beverages remains a pervasive problem internationally [1–3]. Selection of large portion sizes may be an important factor contributing to increases in food and beverage consumption and hence total energy intakes [4], increasing risk of weight gain and chronic disease [5–8]. Interventions to reduce portion sizes typically address the issue in one of two ways. The first is an individual-level approach that conceives of dietary intake as a modifiable behavior under conscious control, and thereby seeks to ameliorate individuals’ dietary choices through downstream educational, motivational and/or behavioral interventions [9]. The second is a multi-level socioecological approach that regards individuals’ eating patterns as embedded within a broader social, cultural, economic and political context [10]. Socioecological interventions to improve dietary intake span multiple levels of influence, from the proximal individual-level to the more distal policy-level, but crucially focus on ameliorating contextual conditions through more distal upstream macro-environmental policy and normative change.

The idea that using smaller sized dishware might nudge individuals to eat less is consistent with individual-level behavioral approaches. The rationale for using smaller plates is informed by the Delboeuf visual illusion, whereby humans perceive a circle surrounded by a small circle as larger compared to when it is surrounded by a larger circle [11]. Applied to the realm of food, items placed on a large plate might appear to represent a smaller portion. It has therefore been postulated that avoiding oversized dishware could help individuals to self-select smaller food portions. The Cornell Food and Brand Lab was an early proponent of using smaller-sized dishware and of other analogous strategies to reduce food overconsumption [11, 12]. The notion that problems related to overeating might be effectively resolved through reengineering our collective kitchens and dining rooms to support behavior change is certainly compelling, as it invokes relatively small, simple and inexpensive interventions that require little to no upstream or collective social action. But does the evidence support the efficacy of such approaches?

The newly published paper by Kosīte et al. [13] reports findings from a rigorous, pre-registered investigation of the effects of manipulating plate size on total energy intake within a single eating occasion. This study comes against a backdrop of considerable uncertainty regarding the potential effects of dishware size, as systematic reviews have yielded conflicting findings in this respect, include studies of relatively low methodological quality and do not include more recent publications since they are several years out of date. For instance, a 2015 Cochrane review and meta-analysis of 72 randomized controlled trials (RCTs; the meta-analysis included 86 independent comparisons from 58 studies) found small to moderate effects of portion, package, individual unit or dishware size on food selection and consumption, with no evidence that findings differed for the subgroup of 12 studies that specifically manipulated dishware size [14]. However, ratings of the quality of the evidence ranged from very low to moderate due to serious concerns around unclear and/or incomplete reporting of study methodologies and procedures. Robinson et al. [15] conversely found no consistent effects of manipulating dishware size on food intake in randomized and non-randomized studies. In their 2016 review of randomized and non-randomized studies, Holden et al. [16] found large effects of manipulating plate size when participants were unaware that they were participating in a food study and when food was self-served, but no effects when portion size was held constant. The latter findings suggest that the effects observed may have been partially mediated by a portion size effect.

This lack of clarity in findings from systematic reviews is compounded by the fact that all three reviews included multiple studies authored by the Cornell Food and Brand Lab, which has come under scrutiny for possible scientific misconduct and recently retracted or corrected numerous publications. Although none of the retracted articles were included in the reviews, there is concern that these studies often reported large effect sizes when others found no evidence of effects [15, 17]. Notably, 13 of the 72 articles in the Cochrane review originated from the Food and Brand Lab, all of which were rated at high or unclear risk of bias. The latter was due to incomplete study reporting and subsequent failure to supply missing data [14].

Given this context, the current well-conducted RCT by Kosīte et al. [13] is very timely and important. The study was pre-registered, included an evidence-informed a priori sample size calculation, adhered to rigorous methodologic protocols, studied a diverse sample of participants, and was conducted in a naturalistic setting whereby participants were unaware of the true purpose of the study. The authors found no clear evidence of a difference in the primary outcome of caloric intake between groups randomized to serve themselves food onto a smaller or a larger plate, with a very small standardized mean difference of 0.07 between groups [18], or the equivalent of 19.2 kcals. Given the wide 95% confidence intervals (− 76.5 to 115.0), the authors nevertheless caution that a small to medium effect may potentially exist in either direction. There was no effect modification according to several potential effect modifiers, including executive function, sensitivity to peripheral cues or indicators of socioeconomic status; nor was there evidence that aspects of the meal such as the number of food servings, eating rate or bite size differed between groups.

So how important are these results? Although the study was rigorous and the overall differences between groups was very small and not statistically significant, the authors duly caution that the observed wide confidence intervals do not rule out the possibility of a small to medium effect in either direction. Thus, further high quality studies are needed, as no single study can ever offer a definitive answer to any scientific question due to unavoidable biases that exist even within well-designed RCTs [19]. This study therefore serves as an opportunity to call for rigor in scientific research and highlights how low quality studies can undermine progress. If all previous studies had been as rigorous as this one, meta-analyses of similarly well-conducted RCTs may have provided much clearer evidence of the impacts of manipulating dishware size on food consumption by now.

Scientists will undoubtedly disagree as to the probative value of further studies in this area, and the nature of any studies that might be undertaken, based on whether they subscribe to an individual-level behavioral or a multi-level socioecological paradigm of health. For instance, some might consider it worthwhile to examine whether manipulating dishware size might influence food consumption under certain conditions; such as if individuals eat when distracted, when using certain types of dishware (e.g. bowls vs plates), when food is self-served rather than pre-portioned, if meals entail amorphous mixed dishes or discrete snack items, among distinct population subgroups or at certain times of day. Clearly an endless array of investigations could be pursued at considerable time, effort and expense. However, we would suggest that key questions might instead relate to whether manipulating dishware size has value at a broader population-level; such as one aspect of broader multi-level policy interventions within publicly funded institutions (e.g. schools, hospitals). Such studies would entail long-term investigations of the effects of manipulating dishware size on intake of commonly consumed foods across multiple meals and days, and would need to be performed in large, diverse, population-based samples under free-living conditions.

In our estimation, the notion that one or even a series of small behavioral nudges could resolve problems related to overconsumption of foods and beverages belies a relatively simplistic understanding of the primary drivers of food consumption. The science of nudging intimates that individuals make ‘poor dietary choices,’ and that these poor choices can be relatively easily corrected, downplaying the broader contextual issues at play. A more comprehensive perspective regards food consumption as a complex practice shaped by the opportunities and constraints imposed by individuals’ broader social, cultural, economic and political contexts. For example, individuals who are working multiple low-wage jobs may use food to cope with the stress generated by difficult employment conditions, while individuals who are experiencing food insecurity may eat more when food is abundant given future expectations of scarcity [20]. People in difficult life circumstances cannot simply be nudged to less, as eating patterns are enmeshed within their broader social, cultural, economic and political contexts [21, 22]. Thus behavioral nudges are on their own unlikely to override more powerful contextual drivers of food consumption, particularly over the longer-term.

Governments have many policy levers available to them, however their resources are not infinite. An excessive focus on individual-level behavioral interventions risks crowding out more powerful interventions that address the fundamental causes of overeating rooted in social, cultural, economic and political contexts. Although behavioral nudges need not be abandoned and can be integrated within more comprehensive multi-level socioecological approaches, their potential impact may have been exaggerated in the past. Given the poor quality of much of the prior research pertaining to the effects of manipulating dishware size and the null findings reported herein, there is at present no substantive evidence upon which to recommend the use of smaller dishware to curb overconsumption. Fortunately, as evidenced by Kosīte et al’s work, it is possible to conduct high-quality studies in this respect. We would therefore encourage future high-quality RCTs to establish whether reducing dishware size and other behavioral nudges might offer an effective complement to more comprehensive multi-level interventions to reduce overconsumption of foods and beverages at a population-level.

## Data Availability

Not applicable
